# Neuroinflammaging: A Tight Line Between Normal Aging and Age-Related Neurodegenerative Disorders

**DOI:** 10.14336/AD.2023.1001

**Published:** 2024-08-01

**Authors:** Luca Soraci, Andrea Corsonello, Ersilia Paparazzo, Alberto Montesanto, Francesco Piacenza, Fabiola Olivieri, Maria Elsa Gambuzza, Edlin Villalta Savedra, Silvia Marino, Fabrizia Lattanzio, Leonardo Biscetti

**Affiliations:** ^1^Unit of Geriatric Medicine, Italian National Research Center of Aging (IRCCS INRCA), Cosenza, Italy.; ^2^Department of Pharmacy, Health and Nutritional Sciences, University of Calabria, Rende, Italy.; ^3^Department of Biology, Ecology and Earth Sciences, University of Calabria, Rende, Italy.; ^4^Advanced Technology Center for Aging Research, Italian National Research Center of Aging (IRCCS INRCA), IRCCS INRCA, Ancona, Italy.; ^5^Department of Clinical and Molecular Sciences, Università Politecnica delle Marche, Ancona, Italy.; ^6^Clinic of Laboratory and Precision Medicine, Italian National Research Center of Aging (IRCCS INRCA), Ancona, Italy.; ^7^Territorial Office of Messina, Italian Ministry of Health, Messina, Italy.; ^8^Independent Researcher, Cosenza, Italy.; ^9^IRCCS Centro Neurolesi "Bonino-Pulejo”, Messina, Italy.; ^10^Italian National Research Center of Aging (IRCCS INRCA), Ancona, Italy.; ^11^Section of Neurology, Italian National Research Center on Aging (IRCCS INRCA), Ancona, Italy.

**Keywords:** Neuroinflammation, innate immunity, epigenetic changes, inflammatory cytokines, epigenome dynamics

## Abstract

Aging in the healthy brain is characterized by a low-grade, chronic, and sterile inflammatory process known as neuroinflammaging. This condition, mainly consisting in an up-regulation of the inflammatory response at the brain level, contributes to the pathogenesis of age-related neurodegenerative disorders. Development of this proinflammatory state involves the interaction between genetic and environmental factors, able to induce age-related epigenetic modifications. Indeed, the exposure to environmental compounds, drugs, and infections, can contribute to epigenetic modifications of DNA methylome, histone fold proteins, and nucleosome positioning, leading to epigenetic modulation of neuroinflammatory responses. Furthermore, some epigenetic modifiers, which combine and interact during the life course, can contribute to modeling of epigenome dynamics to sustain, or dampen the neuroinflammatory phenotype. The aim of this review is to summarize current knowledge about neuroinflammaging with a particular focus on epigenetic mechanisms underlying the onset and progression of neuroinflammatory cascades in the central nervous system; furthermore, we describe some diagnostic biomarkers that may contribute to increase diagnostic accuracy and help tailor therapeutic strategies in patients with neurodegenerative diseases.

## 1. Introduction

Inflammaging was originally defined by Franceschi et al. as the chronic, sterile, and low-grade systemic inflammation that typically characterizes aging [1]. In the last two decades, the concept of inflammaging has been becoming more and more relevant in geriatric research field, since inflammaging level is now recognized as the main risk factor for the development of the most common age-related diseases [2].

This proinflammatory state can be triggered and sustained by different stimuli, including exogenous pathogens, endogenous cell debris, nutrients, and gut microbiota; the abnormal activation of innate immunity cells, primarily macrophages, and the dysregulation of T-cell responses in the context of adaptive immunity concur to propagation and progression of inflammaging [2-4]. Another culprit of inflammaging is the increased burden of senescent cells acquiring a proinflammatory secretory phenotype (SASP) [1, 2]. Starting from this perspective, the current approach by geroscience suggests strict interconnections between aging, inflammaging and age-related diseases.

Also at brain level, normal ageing is frequently accompanied by a low-grade, chronic, and sterile inflammatory process, defined as “neuroinflammaging”, which may predispose to the onset and progression of several cerebrovascular and neurodegenerative diseases [5]; indeed, brain aging is associated with an up-regulation of proinflammatory pathways within the resident glial cells, such as astrocytes and microglia, which, despite their brain-specific names, are close relatives of the macrophages [6-8]. Furthermore, the chronic exposition to inflammatory stimuli along life may damage, at least partially, the blood-brain barrier (BBB) integrity [9], thus allowing the transposition of circulating peripheral immune cells and cytokines into the central nervous system (CNS), finally contributing to the neuroinflammaging process. Increased glial levels of pro-inflammatory cytokines including interleukin 1β (IL-1β) and tumor necrosis factor- α (TNF-α) were already related with the onset and progression of cerebrovascular, autoimmune and neurodegenerative disorders [10], at least in particularly vulnerable individuals.

In this view, both inflammaging and neuroinflammaging can be seen as borderline conditions between normal aging and age-related diseases, so that one of the cutting-edge challenges is to identify inflammaging cut-off level associated with pathological conditions. In this framework, it is relevant to disentangle the molecular pathways that fuel inflammaging, focusing on genetic, epigenetic, and environmental factors and their complex interactions [7, 11, 12].

To improve the understanding of factors promoting the onset of neuroinflammation in age-related diseases and to prevent and/or reduce their occurrence in human population, current studies are specifically exploring the putative links between epigenetic modifications and the activation of neuroinflammatory pathways during aging.

Epigenetic modifications are reversible mechanisms of DNA regulation that depend on chemical modifications of DNA and histone proteins, without changes in DNA sequence [13]. With advancing age, chronic exposure to intra- and extracellular agents including diet, drugs, and stressors progressively shape the epigenetic machinery [14-16], thus contributing to sustain or dampen the aging-related neuroinflammatory phenotype [17].

Overall, the aim of this review is to summarize the current knowledge about neuroinflammaging with a particular focus on epigenetic mechanisms, underlying the onset and progression of neuroinflammatory cascades in the CNS; furthermore, we will try to furnish a solid platform for future research, highlighting some promising diagnostic biomarkers that may contribute to increase diagnostic accuracy and help tailoring targeted interventions aimed to modulate epigenetic mechanisms to counteract neuroinflammatory responses.

## 2. Neuroinflammaging in the aged brain

### 2.1. Central immune dysfunction in neuroinflammaging

Normal aging is associated with morphological and functional senescence of both central and peripheral immune cells, which stop growing and undergo many phenotypic and functional alterations, primarily the acquisition of SASP. This intrinsic alteration of the immune system, a process termed “*immunosenescence”*, is mainly characterized by a progressive decline of innate immune responses, as well as a progressive imbalance of cytokine production towards the development of a pro-inflammatory phenotype [18]. These aging-related changes involve both CNS and peripheral immune cells [19-21]. In contrast to neurons which decrease with advancing age, the CNS immune cells represented by glial cells experience a progressive increase in number and cellular immunoreactivity [20].

Among the glial cells, microglia and astrocytes play specific roles in CNS homeostasis, contributing to regulate immune response, BBB maintenance and synaptic development and plasticity [20] .

In elderly human brain, dystrophic microglia, which show the classical amoeboid cell morphology characteristic of activated microglia, express inflammatory signals that have been associated with both neuroinflammation and neurodegeneration [8, 22]; similarly, senescent astrocytes acquire morphological and detrimental signatures, that impair their ability to properly maintain a healthy CNS environment and enhance the expression of proinflammatory molecules [23]. Altogether, these events may contribute to the occurrence of neurodegenerative diseases characterized by the accumulation of misfolding proteins in the brain, including alpha-synuclein (which is typically involved in Parkinson disease (PD)), and tau.

To this regard, it has been previously suggested that senescent glial cells may contribute to the pathology of Parkinson’s disease in both mice and humans [24]. Additionally, recent findings have shown that continuous clearance of p16Ink4a-expressing senescent cells before disease onset in a model of aggressive tauopathy has a marked effect on various aspects of disease progression, including gliosis, neurofibrillary tangles (NFT) formation, neurodegeneration, and cognitive decline [25]. Senescent cell clearance has also been found to have a notable effect on the accumulation of phosphorylated tau protein in both the soluble and insoluble fractions [25]. Accordingly, a prompt clearance intervention of accumulated senescent cells might be a viable therapeutic strategy to counteract age-related cognitive decline [26].

Regarding the molecular pathways involved in microglia and astrocytes activation, as well as in cell senescence, it is noteworthy that these glial cells release a number of immunomodulators and express specific types of immune receptors, known as pattern recognition receptors (PRRs), such as Toll-like receptors (TLRs), absent in melanoma 2 (AIM2) like receptors (ALRs) and NOD-like receptors pyrin containing proteins (NLRPs) [27, 28]. Microglial PRRs can be stimulated after binding to both molecular motifs belonging to exogenous pathogens, also known as pathogen-associated molecular patterns (PAMPs), and endogenous compounds released by damaged cells, also known as damage-associated molecular patterns (DAMPs) [29].

Senescent glial cells are characterized by SASP with overexpression of some TLR genes, such as *tlr2, tlr4,* and *tlr5,* as well as inflammasome-associated genes, such as *cas-1, IL-18* and *IL-1* [30-32]. Furthermore, microglia is involved in physiological neuroinflammatory responses in healthy brain; indeed, both aging and stress induce priming of microglia, characterized by increased and prolonged expression of some TLRs as well as over-reactivity to proinflammatory stimuli [33]. More specifically, activation of TLR2 and TLR4 on the surface of microglia enhances phagocytosis of damaged neurons, contributing substantially to neuronal loss during neuroinflammation [34]. Similarly, a prolonged stimulation of TLR2, TLR4, and TLR9 can further enhance the inflammatory response and production of neurotoxic molecules [35, 36]. Also, astrocytes express significant levels of several TLRs, such as TLR1-5 and TLR9; while TLR3 activation drives anti-inflammatory modulation, stimulation of TLR2 and TLR4 is most frequently associated with proinflammatory responses [37, 38].

In any case, activation of TLR-dependent pathways is regulated by two main adaptor proteins, including the myeloid differentiation primary response 88 factor (MyD88), and the Toll/IL-1R domain-containing adaptor-inducing IFN-β (TRIF) [39]. All TLRs signal via the MyD88-dependent pathway, except for TLR3, which signals exclusively via TRIF.

**Figure 1.**
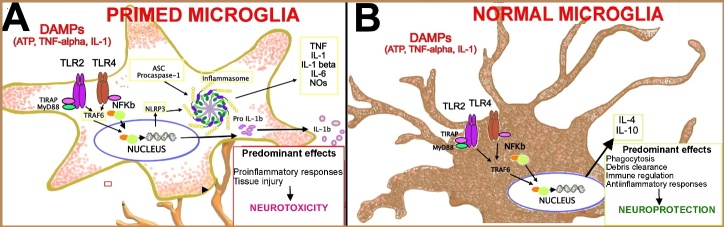
**Effects of TLR-mediated responses induced by endogenous stimuli, in primed and normal microglia**. (**A**) Inflammatory profile in aged microglia (*primed microglia*) activation. In aged microglia, which show predominantly the amoeboid cell morphology characteristic of activated immune cells, NLRP3 priming occurs through two distinct steps. First, NF-κB-activating stimuli, as well as DAMPs, also including ATP, TNF, and IL-1, bind to TLR4, inducing increased expression of *NLRP3* and *IL1B*, which induce inflammasome assembly. Additionally, priming immediately licenses NLRP3, by inducing its deubiquitination. The adaptor protein ASC must become linearly ubiquitinated and phosphorylated for inflammasome assembly to occur. The formation of the NLRP3 inflammasome results in the activation of caspase-1, through the self-cleavage of procaspase-1. Activated caspase-1 causes the maturation of IL-1β and triggers inflammatory response, via the production of large amounts of proinflammatory cytokines, chemokines, and reactive species. (**B**) Anti-inflammatory profile in young microglia activation. The activation of microglia with anti-inflammatory profile promotes the release of IL-6 and IL-10, with neuroprotective effects.

More specifically, MyD88 promotes the induction of inflammatory pathways through IL-1 receptor associated kinases (IRAKs), leading then to the induction of, mitogen-activated protein kinases (MAPKs), and activator protein 1 (AP1) [40]. This signaling leads to the activation of nuclear factor kappa-light-chain-enhancer of activated B cells *(*NFκB) and interferon regulator factors (IRFs), which regulate the expression of proinflammatory cytokines (i.e., pro-IL-1β and pro-IL-18 by NF- kB, and IFN I by IRFs, respectively).

### 2.2 PRRs and Inflammasome Activation in the aged brain

NF-κB is a central mediator of the priming signal of NLR family pyrin domain containing 3 (NLRP3) inflammasome activation. It acts by inducing the transcriptional expression of NLRP3, an intracellular multimeric protein complexes that in turn activate inflammatory caspase-1 (Cas-1), leading to pro-inflammatory cytokine production (IL-1β and IL-18) [41].

NLRP3 complex is formed by a specific sensor protein, an adaptor, and a zymogen procaspase, the pro-Cas-1. Assembly of NLRP3 inflammasome is favored by activation of TLR4 [42]. More specifically, activated TLRs recruit adaptor molecule apoptosis-associated speck-like protein containing a CARD (ASC) and pro-Cas-1 which stimulate the production of pro-inflammatory cytokines. In turn, pro-inflammatory cytokines may further stimulate TLRs and NLRP3 inflammasome activation, thus creating a feed-forward loop that increases the neuroinflammatory responses [41]. Interaction between TLRs and NLRP3 in primed and normal microglia is shown in Figure 1.

NLRP3 inflammasome plays a crucial role in host immune defenses against bacterial, fungal, and viral infections [43-45]. In the neuroinflammaging context, a typical dysregulation of TLR-mediated NLRP3 inflammasome activation within the CNS frequently occurs [41], thus contributing to increase the risk of developing several age-related and neurodegenerative diseases [46, 47]. In aging brain, inflammasome activation may be triggered by local microenviroment changes associated with priming of microglia [48, 49]. Primed glial cells are more susceptible to adopting a pro-inflammatory state that also primes the cells for inflammasome activation. Additionally, aging-induced accumulation of lipofuscin impairs the neuroprotective potential of microglia and contribute to age-related pathology [50].

### 2.3 AGE-RAGE activation in the aging brain

Among factors putatively involved in age- related brain changes, we have to consider also advanced glycation end-products (AGEs; e.g., glyoxal, methylglyoxal or carboxymethyl-lysine).

AGEs are an heterogenous group of toxic compounds synthesized in the body through both exogenous and endogenous pathways. AGEs are known to covalently modify proteins bringing about loss of functional alteration in the proteins. AGEs also interact with their receptors, called RAGE, which are present also in soluble form (sRAGE). Previously, AGE-RAGE axis has long been associated with aging and various human age-related diseases including diabetes, obesity and cardiovascular diseases. Recent developments have revealed the involvement of AGE-RAGE axis in the onset of neurodegeneration through several mechanisms, including disruption of BBB, and neuroinflammation [51]. AGEs-RAGE interaction modulates intracellular signaling activating NF-kB. This inflammatory signaling cascade is associated with various neurological diseases, including Alzheimer's disease (AD), secondary effects of traumatic brain injury (TBI), amyotrophic lateral sclerosis (ALS), and diabetic neuropathy, and other AGE-related diseases, including diabetes and atherosclerosis [52].

Commonly AGE accumulation is observed in neural cells and tissues during aging, and some pre-clinical data suggest that the extent of such accumulation may be related with the presence of memory impairment and AD-like pathologic changes [53]. Overall, many neuropathological and biochemical aspects of AD might be explained by AGEs, including widespread protein crosslinking, glial activation of oxidative stress, neuroinflammaging and, finally, neuronal cell death.

### 2.4 Neuroinflammaging and Epigenetic Changes

Neuroinflammaging is strictly connected with age-related epigenetic changes. Epigenetic changes consist in modifications affecting the gene accessibility to DNA-binding and the molecular components involved in the transcription machinery modulation. More specifically, these epigenetic modifications induce changes of the DNA structure after its replication, and/or post-translational alterations of DNA-associated proteins [54, 55]. Epigenetic changes appear to be promoted by brain and peripheral pro-inflammatory stimuli, capable of triggering extensive epigenetic reprogramming in glial responses [56].

Despite many of these epigenetic changes are considered an unavoidable event due to genetic and stochastic factors, they can also arise as a result of exogenous stimuli that may then contribute to neuroinflammaging [11, 57]. Pioneering studies have shown that the innate immune system also exhibits adaptive characteristics, a property recently termed “*trained immunity”* [58]. Trained immune cells are more susceptible to undergo epigenetic changes and develop neuroinflammatory responses [58].

**Figure 2.**
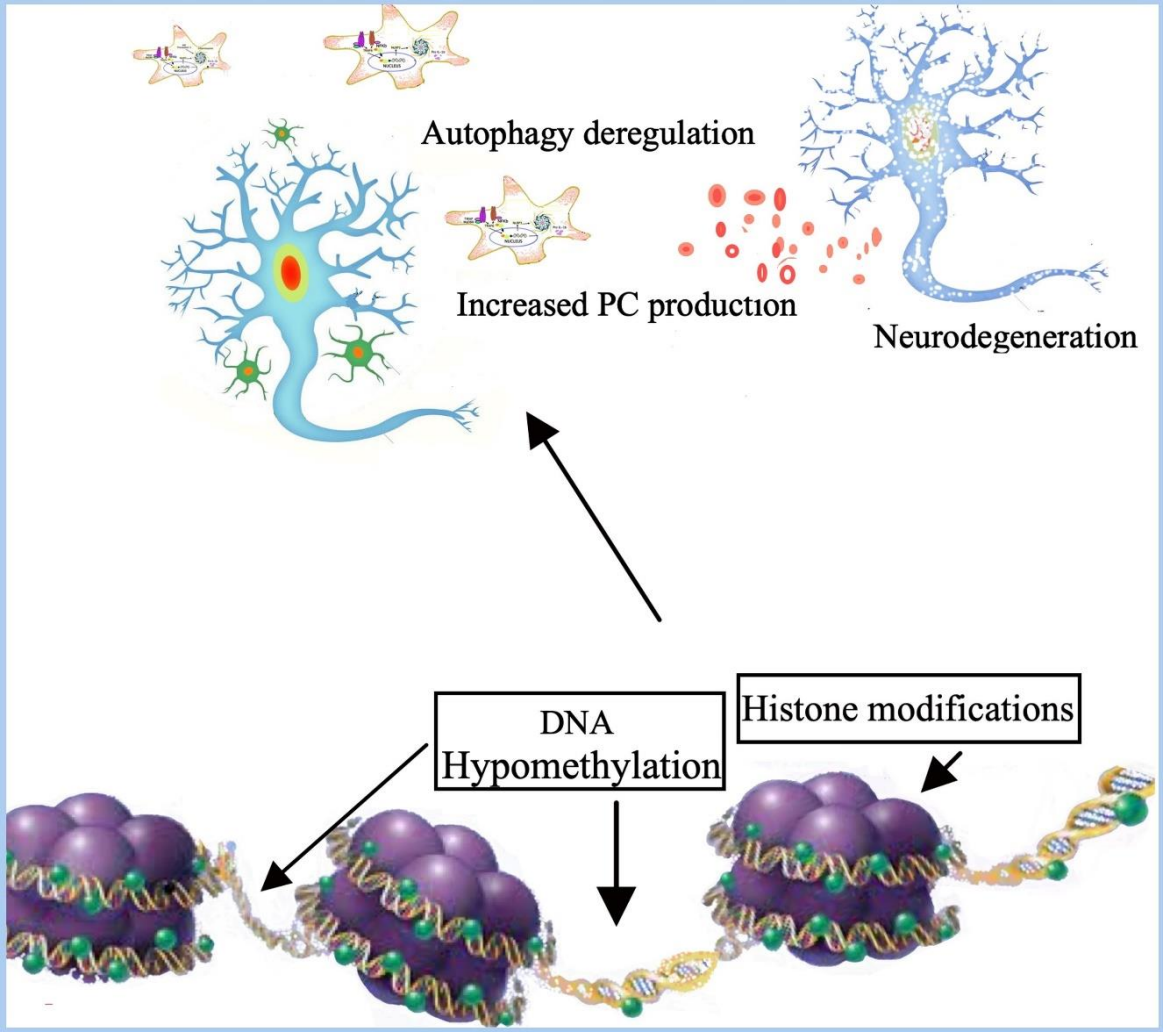
**Epigenetic changes and neuroinflammaging**. Altogether, aging appears associated with a gradual deterioration of epigenomic markers, such as altered methylomic patterns, mainly characterized by a global hypomethylation in the DNA genome and specific histone modifications. In the aged brain DNA hypomethylation and histone modifications induce a progressive autophagy deregulation, mainly consisting in an increased number of dystrophic microglia and astrocytes, which show the classical amoeboid cell morphology characteristic of activated phagocytic cells, with the development of a neuroinflammatory profile, that predisposes to neurodegenerative conditions.

Epigenetic mechanisms mainly involved in brain inflammaging responses include DNA methylation, histone proteins modifications, chromatin remodeling and changes in gene expression induced by non-coding RNA, *i.e.* microRNAs (miRNAs)[59-61] (Fig. 2).

DNA methylation is an epigenetic mechanism of gene repression through the addition of methyl groups to the aromatic ring of CpG dinucleotides that impairs interactions with transcriptional regulators [57]. A global decline in DNA methylation patterns was observed in the senescent cells compared to the young ones and it was associated with a decrease in brain function and memory loss [62]. Previous studies have reported a consistent increase in age-related DNA hypomethylation, that induces the generation of unmethylated DNA fragments, which can act as self-derived proinflammatory stimuli [63-65]. Recent studies have also shown that modifications in DNA methylation are often associated with NLRP3 inflammasome-related neuroinflammatory processes [66].

Among the whole genome sequences, particular attention might be warranted for epigenetic alterations of transposable elements (TEs). TEs are DNA sequences that are able to change their position within a genome [67]. They are highly repetitive DNA sequences that make up more than 50% of the human genome and contain approximately 52% of all CpG dinucleotides [68]. Consequently, methylation at TEs is considered an indicator of global DNA methylation. DNA methylation is arguably the most extensively studied regulator of TE activity. It is associated with the repression of TEs, serving to limit their genotoxic potential. Conversely, the removal of DNA methylation is associated with a surge in TE expression [69].

In recent years, TEs, which were once considered 'junk' DNA, have garnered increasing interest as potential contributors to neurological disorders. Aging represents the primary risk factor for most neurological diseases, and several repressive mechanisms of TEs, such as heterochromatinization, become less effective with age [69]. Consequently, heterochromatin relaxation leading to TE de-repression has been observed in various models of neurodegeneration and neurological disorders. Additionally, evidence suggests that certain pathogenic proteins involved in neurodegeneration (e.g., tau, TDP-43) may regulate the expression of TEs [70]. The detrimental consequences of TE activation are not yet fully understood, but they could include DNA damage, genomic instability, altered host gene expression, and/or neuroinflammation, which are common hallmarks of neurodegeneration and aging. Thus, TEs may represent an overlooked pathogenic factor in both brain aging and neurodegeneration, and inhibiting their activity could potentially prevent certain pathological effects, making TEs novel targets for neuroprotection.

While several studies have highlighted the role of TEs in certain diseases [71], only a limited number have investigated their relationship with neurodegeneration and neuroinflammation. Nevertheless, accumulating evidence suggests that retrotransposition (a type of transposition based on the transcription of some TEs in RNA followed by retrotransciption into DNA and subsequent transposition of DNA sequences), plays a significant role in shaping somatic mosaicism, contributing to the functional specification of brain cells [72, 73]. For example, it has been reported that the transposition of long interspersed element (LINE)-1 sequences may be involved in neuron differentiation during brain development [74]. Moreover, this sequence exhibits autonomous retrotransposition activity in neural rat progenitor cells from the hippocampus, human fetal brain, and human embryonic stem cells [75, 76]. Therefore, it has been hypothesized that aberrant epigenetic regulation of TEs may play a crucial role in the development of neurological disorders [70].

Histone modifications represent further key epigenetic regulators that control chromatin structure and gene transcription. The fundamental unit of chromatin, termed “*nucleosome”,* consists in a histone octamer core, composed by two copies of H2A, H2B, H3 and H4 histones, and wrapped by a portion of DNA sequence containing about 147 bp [77]. Similarly to DNA methylation, post-translational histone modifications do not affect DNA nucleotide sequence, despite they can modify its availability to the transcriptional machinery [77].

In normal conditions, the large number of histone changes tightly regulates the chromatin structure and counteracts the harmful effects induced by histone accumulation, that can occur during the slowing or stopping of DNA replication and may lead to genomic instability [78].

During the aging process, the main effects induced by histone changes consist in aberrant histone modifications, histone loss and/or repositioning, accumulations of histone variants and altered chromatin accessibility [78, 79]. Age-related deregulation of cellular autophagy has been shown to be associated with an increased acetylation of histone H3 and changes in methyltransferase G9a activation [80]; similarly, alterations in global methylation of H3 and H4 have been described during aging and neurodegeneration [81-83].

Altogether, histone acetylation and methylation have been shown to play a critical role in activation or repression of proinflammatory cytokine transcription [84].

Like acetylation, histone phosphorylation is generally associated with transcriptional activation, and contributes to the DNA damage repair, the control of chromatin structure during mitosis and meiosis, and the regulation of transcriptional activity [85].

In activated microglia, elevated histone H3 phospho (Ser10)-acetylation (Lys14) enhances the expression of pro-inflammatory genes, such as *IL-1β*, *IL-6*, *TNF-α*, *iNOS*, and *c-Fos* [86].

Histone ubiquitination, consisting in the post-translational transfer of ubiquitin to the histone core proteins, such as H2A and H2B, differs substantially from the other epigenetic mechanisms, because it consists in a very large modification; while H2A ubiquination mostly leads to gene silencing, H2B ubiquitination has been linked to gene activation linked to memory function[87, 88]. While histone H2B deubiquitination mediates the repression of the inflammatory response, histone ubiquination plays an important role in innate immune response and in the selective expression of proinflammatory cytokines [89].

MicroRNAs represent another important class of epigenetic regulator. MicroRNA are the shortest and non-coding ssRNAs (19-22 nucleotides) that regulate gene expression through the binding to sequence motifs located within the 3’ untranslated region of mRNA transcripts; miRNAs are capable of repressing the mRNA translation and/or inducing its degradation. The regulation of expression of miRNAs in aging has become an emerging field of interest in the last decades [90]. Recently, increased miRNA expression in human peripheral blood was correlated with the development or progression of aging-associated diseases [91]; furthermore, the age-related increase in immunomodulatory miRNA-29 has been reported to be associated with the expression of an inflammatory profile of aged microglia [92].

Another relevant microRNA in this framework is miR-146a, that stands out due to the increasing number of papers in recent literature focused on its mechanistic involvement in neurological diseases. MiR-146a polymorphisms are associated with the risk of neurological disease and alterations in miR-146a expression levels are crucial events in the pathogenesis of numerous neurological diseases [93].

In animal models, microglia-specific miR-146a overexpression reduced cognitive deficits in learning and memory, attenuating neuroinflammation, reducing Aβ levels, and ameliorating plaque-associated neuritic pathology. In addition, miR-146a was able to switch the microglial phenotype, reducing pro-inflammatory cytokines and enhancing phagocytic function to protect neurons [94]. Notably, miR-146a is able to target a variety of molecules belonging to the NF-κB/NLRP3 pathways [95].

Taken together, the above findings suggest that a dysregulation of specific miRNAs could further contribute to over activation of microglia and chronic neuroinflammation [96].

### 2.5 Molecular and environmental basis of neuroinflammaging

Neuroinflammaging has recently been considered as a multifactorial pathological process, resulting from the interaction of genetic, molecular, and environmental factors [97].

Among the molecular compounds belonging to brain microenvironment and considered endogenous risk factors, the toxins originated from brain cell death play a very important role as neuroinflammatory factors. In this context, glial cells play a fundamental role, as they are able to detect foreign material in the homeostatic neural environment and to promote the production of cytokines, nitric oxide, and other reactive oxygen species (ROS), which may adversely impact adjacent neurons [98]. This apparently simple inflammatory response can become hyper stimulated in the context of the aging-induced imbalance between up-regulated proinflammatory and down-regulated anti-inflammatory responses [30].

The persistent stimulation of glial cells and neurons, which enhances the production of neurotoxic factors, leads to the accumulation of inflammasome polymers, such IL-1, TNF-α, and IL-6, sphingolipids, and neurothrophins, that can enhance neuroinflammation through detrimental astrocytic signaling pathways [99]. Another endogenous factor contributing to neuroinflammaging is represented by mitochondrial dysfunction; with advancing age, mitochondrial ATP production and membrane potential decrease, while oxidative stress within mitochondria tend to increase. Accumulation of ROS further induces biochemical and physiological alterations ultimately leading to mitochondrial and cell damage. Altogether, these molecular changes stimulate all forms of programmed cell deaths, including apoptosis, necroptosis, and pyroptosis, which are involved in the development and progression of the main neuroinflammatory and neurodegenerative pathologies [100, 101].

Along with endogenous factors, several exogenous and environmental agents are implicated in the neuroinflammaging process [102]; diet, alcohol, smoke, air pollutants, drugs, chemical compounds, infectious agents, as well as psychological and social stressors, can represent an external stress For the over-reactive aged brain immune system.

Among the inhaled pollutants, agriculture contaminants appear to contribute to pro-inflammatory phenotype acquirement and ROS generation in glial cells of both affected individuals and animal models [102-104]. Among the chemical neuroinflammation inducers, the prophylactic use of the reversible acetylcholinesterase inhibitor pyridostigmine bromide, in association to the stress of combat, have been identified as two potential causative factors of the multi-symptom illness affecting many American Gulf War veterans, and essentially characterized by cytokine-mediated neuroinflammation [105]. The deleterious effects of pyridostigmine bromide have been also confirmed by further studies on rat models, where it showed to elicit progressive and chronic impairments in the cholinergic anti-inflammatory pathway in the prefrontal cortex and hippocampus [106].

Nutritional regimens have also shown to affect inflammaging and epigenetics, as also confirmed by the anti-inflammatory properties of caloric restriction diets, which induce epigenetic downregulation of NF-κb and AP-1, by increasing the deacetylation of histones by the deacetylase Sirtuin 1 (SIRT1) [107, 108].

Finally, the chronic exposure to addictive compounds such as alcohol, nicotine, cocaine, and cannabinoids, can enhance the release of proinflammatory cytokines by microglia and astrocyte [109] and can disrupt neuroimmune signaling thus increasing the risk for neurotoxicity [109].

Altogether, different lifestyles and environmental stress factors can induce cumulative effects with advancing age and lead to dysregulation of inflammatory responses and perturbations of homeostatic mechanisms. These cumulative stressors can affect the neuroimmune axis, leading to chronic neuroinflammation, that may manifest with nonspecific symptoms (e.g. lethargy, anhedonia, anorexia, depression and cognitive dysfunction); these manifestations are some of the features of “sickness behavior”, a condition that in geriatric people is often overlooked [110].

**Figure 3.**
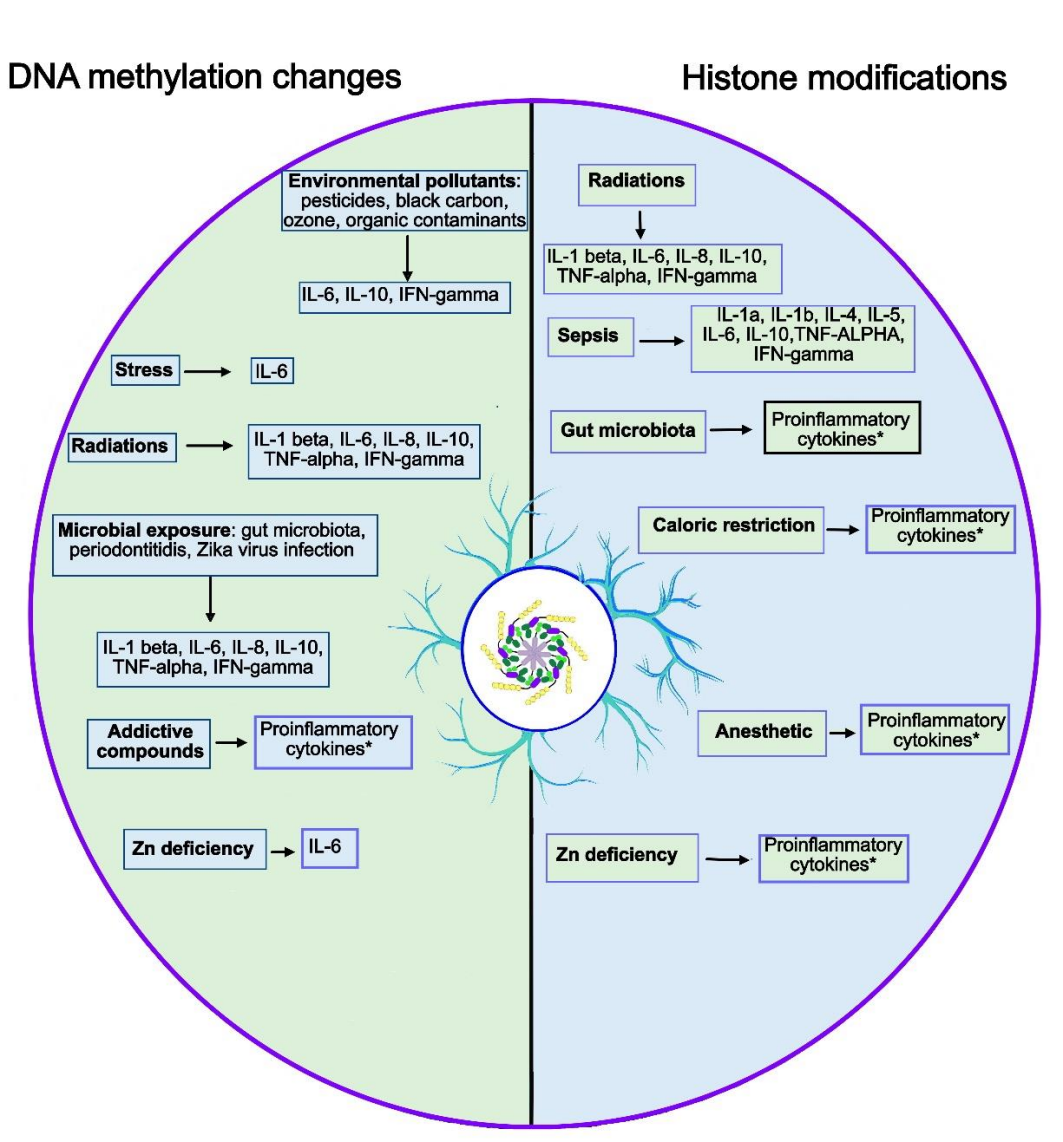
**Epigenetics of neuroinflammaging and environmental factors**. During aging, several exogenous stimuli can further increase various epigenetic alterations, mainly including DNA methylation changes and histone modifications. These aberrant epigenetic processes can promote a neuroinflammatory status predisposing to neurodegeneration. *”Proinflammatory cytokines” indicates an increase in proinflammatory markers without a direct association between the single stimulus and the relative specific cytokines.

Altogether, as represented in Figure 3, the above-described exogenous stimuli, can cooperate with each other, thus contributing to accelerated neuroinflammatory and neurodegenerative processes.

## 3. The Link Between Neuroinflammaging and neurodegeneration

### 3.1 Epigenetic and environmental basis of neuroinflammaging and neurodegeneration

Current evidence suggests that age-induced proinflammatory changes in healthy brain may favor chronic disease development in predisposed individuals, thus tracing a tight link between neuroinflammaging and neurodegeneration [5, 111].

In this regard, serum concentrations of some inflammatory cytokines, such as IL-1β, TNF-α, and IL-6, increase with advancing age [112] and are highly expressed in patients with Alzheimer's disease (AD) or depression [113]. Furthermore, astroglia over-reactivity with perturbations of their cytokine profiles inside the brain has been associated with the onset and progression of neurodegenerative diseases [114, 115]; indeed, neurofibrillary tangles and senile plaques of patients with AD are associated with increased plasma and brain concentration of microglial proinflammatory mediators which in turn can exacerbate neuronal loss and degeneration [116, 117]. Similarly, evidence of increased proinflammatory activity in the brain and cerebrospinal fluid of Parkinson’s disease (PD) patients emerged from several studies [118, 119], especially in individuals carrying certain genetic mutations [118, 120].

Dysregulation of TLR-mediated inflammatory pathways withing glial cells may also contribute to neurodegeneration. A prolonged activation of TLR2, TLR4 and TLR9 receptors on the surface of microglia enhances proinflammatory states through the production of neurotoxic factors and is associated with the onset of several neurodegenerative diseases, including AD and PD [31, 121]. Similarly, prolonged activation of astrocyte TLR2 and TLR4 has been observed in AD and PD pathology [122, 123]. Furthermore, prolonged stimulation of some TLR is known to lead to NLRP3 inflammasome overactivation, involved in the progression of neurodegenerative and cerebrovascular diseases [124].

Altogether, these data strongly support the existence of a strict connection between immunological dysregulation, both at systemic and CNS level, and neurodegeneration in elderly people.

#### 3.1.1. Epigenetic mechanisms implicated in neuroinflammation and neuordegeneration

Among factors influencing the relationship between neuroinflammaging and neurodegeneration, a pivotal role seems to be played by changes in the epigenetic profiles of trained glial cells [125]. As previously mentioned, the most common epigenetic signatures of neurodegeneration include DNA methylation, histone modification, and miRNA. Age-related neuronal and microglial changes in methylation patterns were recently found to predispose to development of AD [126, 127]; hypomethylation of the promoter of several AD-related genes, including APP, CLU and apolipoprotein E may cause its epigenetic activation and further contribute to neurodegeneration in AD [128, 129]. Similarly, methylation of α synuclein and microtubule-associated tau protein genes may contribute to PD development [130].

Age-related histone modifications have been also related to the onset and progression of several neurodegenerative disorders [131, 132]; in this regard, microglia histone H3 phospho (Ser10)-acetylation (Lys14) phosphorylation has been increasingly found in AD patients [133] and is associated with increased production of IL-1β, IL-6, and TNF-α [134], and NF-κB activity [135]. Another histone modification is H3K27 hyperacetylation, that was recently observed in PD patients and related to impaired sirtuin activity [132] and altered transcription of several PD-related genes, including SNCA (which encodes α synuclein), PARK7 and MAPT [132]. Furthermore, hypophosphorylation of H3 serine 10 and the impaired phosphorylation of H3 serine 1 have been observed in both aging and neurodegeneration and showed to be associated with increased production of proinflammatory mediators [136, 137]. Moreover, the increased H2A phosphorylation observed in hippocampal and cortical astrocytes of AD patients compared to controls suggests a correlation between H2A phosphorylation and astrocyte-specific DNA damage [6].

#### 3.1.2 Telomere shortening, aging and neurodegeneration

Even if telomeres are chromosomal end sequences made of hexameric DNA repeats they occupy an interesting zone spanning the border between genetics and epigenetics [138]. One the one hand, telomeres are sequences of DNA but do not code for any known protein, one the other hand, the length of the telomere repeats is important for telomere functions, and this length can increase or decrease reversibly, like other epigenetic mechanisms. Moreover, telomere length has proven to be an important regulator of gene expression and cellular signaling. Recently, it has also been shown that even before a telomere has become short enough to be uncapped, its shortening can influence gene expression.

Telomere shortening is increasingly recognized as implicated in both neuroinflammaging and neurodegeneration [139, 140]. With advancing age, telomeres progressively shorten because of genetic and environmental factors; telomere shortening can increase genomic instability within microglia and astrocytes, thus contributing to cell death and neurodegeneration. Furthermore, short telomeres were shown to increase the expression of the SASP within glial cells, leading to overproduction of proinflammatory molecules and accelerating cell senescence [141].

Based on the above-mentioned findings, telomere shortening is nowadays considered an age-related mechanism potentially involved in neurodegeneration, as it may exacerbate DNA damage and sensitizes neurons to oxidative stress [142, 143].

Overall, telomere shortening seems to be associated with increased AD risk and disease severity[144]; indeed, among patients with amyloid pathology, telomere shortening was associated with increased chance of developing dementia[145]; this evidence is corroborated by the risk associated with deficiency of telomerases, which have a protective role against neuronal death in β-amyloid pathology; in fact, late generation telomerase-deficient mice tend to develop AD [146]. Telomere shortening has been indeed found in cultured rat microglia [147] and was associated with dementia in AD brain samples [148].

Finally, a recent study has reported a correlation between leukocyte telomere shortening and increased plasma levels of IL-1β and IL-18 in patients with mild cognitive impairment and Alzheimer disease [149]. This latter finding suggests that the link between telomere shortening and AD involves not only immunological glial cells, but also white cells in the periphery. If confirmed by future investigations, this intriguing result could be considered another proof of the systemic immunological dysfunction occurring in the context of AD (we will discuss this issue later in the present review) [150]. These data globally support a strong link between telomere shortening which characterizes aging, and AD development.

The relationship between telomere length and PD, instead, until now, based on the available evidence, results to be less striking, as both longer and shorter telomeres were found in patients with PD compared to healthy controls [151]. However, mice with critically short telomeres express some PD features and tend to develop poor neuromuscular coordination [146]; moreover, activators of telomerases were shown to increase the clearance of α-synuclein and improve motor symptoms in mice with PD [152]. Finally, telomere shortening was reported to induce acceleration of synucleopathy in a mouse model of PD [153].

#### 3.1.3 Environmental factors influencing the risk of neurodegenerative disorders by inducing neuroinflammatory responses

Several environmental factors can exacerbate the age-induced neuroinflammatory responses, thus contributing to increased risk for neurodegenerative diseases in predisposed individuals, for instance by inducing epigenetic modifications or accelerating telomere shortening [154]. In this regard, according to genome wide association studies, several environmental agents like exposure to pesticides, metal, pollution, and diet seem to be pathogenically related to neurodegeneration [155, 156].

Chronic exposure to air pollutants, such as particulate matter, black carbon, ozone, and persistent organic pollutants, has been associated with neurotoxicity via increased production of proinflammatory cytokines, mitochondrial dysfunction, and alteration of DNA methylation patterns within neurons and glial cells [157, 158]; interestingly, sensitivity to air pollutants seemed to be enhanced by genetic factors, as shown among ApoE ɛ4 carriers with AD [159] and leucine-rich repeat kinase (LRRK) carriers with PD [160]. Furthermore, many factors related to social deprivation, including low-quality diet and chronic psychosocial distress, may concur to epigenetic dysregulation of several inflammatory signaling pathways, thus contributing to neurodegenerative disease onset and progression [161].

An accumulating amount of scientific evidence suggests that ionizing radiation exposure may accelerate immune senescence and exacerbate age-induced neuroinflammatory responses, thus favoring the onset of neurodegenerative disorders [162, 163]. Both acute and chronic exposure to ionizing radiations may alter the function of the CNS by increasing oxidative stress, mitochondrial dysfunction, and epigenetic dysregulation with increased production of proinflammatory cytokines, along with acceleration of immune senescence and neuronal death. Furthermore, exposure to ionizing radiation facilitates the misfolding and aggregation of globular proteins [162, 163].

Additionally, many clinical and laboratory studies have recently underlined the role of microbiota in the development of several neurodegenerative diseases [164]. Emerging evidence suggests that gut microbiota, which is influenced by several factors including food and drugs, and epigenetics may directly influence the pathogenesis of AD, by increasing neuroinflammation, oxidative stress, and cytokine storm syndrome [165]. Metabolites produced by the human gut microbiome may also affect the activity of acetylases and methylases, thus inducing histone modifications and DNA methylation, which are commonly found in AD patients [165].

### 3.2 The emerging interplay between the brain-gut axis and the peripheral immune system in the pathogenesis of age-related neurodegenerative disorders

Despite neuroinflammation and neurodegeneration were originally considered as CNS-specific disease processes, increasing evidence is focusing on the potential crosstalk between the CNS, peripheral immune system and gut microbiota in their pathogenesis [166]. Furthermore, the discovery of the immune-brain and gut-brain axes support the potential influence of inflammaging-induced peripheral immune modifications on development of CNS-specific diseases. In this regard, normal aging is characterized by an increased serum level of proinflammatory cytokines, including IL-1, IL-6, TNF-α, and PGE2, which may generate an inflammatory environment for tissue and organs, including the brain [167]. The CNS is extremely responsive to peripheral proinflammatory cytokines which may cross the BBB and act upon the glial cells, thus increasing the expression of SASP and vulnerability to disease [168]. Furthermore, age-related microglial and astrocytic activation leads to release of proinflammatory cytokines that may get into the peripheral circulation through the BBB, thereby recruiting peripheral immune cells which exacerbate the neuroinflammatory cascades [169]. Indeed, peripheral adaptive immune responses are dysregulated in aged individuals, as a consequence of decrease in circulating B cells and naïve T cells, while differentiated T lymphocytes tend to rise [21] with increase in Th1/Th2 ratio. More specifically, increase in differentiated Th1 and Th17 cells may directly enhance neuroinflammation by increased release of pro-inflammatory molecules including IL-1, IL-6, IL-17, TNF-α, and IFN-γ [170]; moreover, these cells exacerbate microglia-mediated neurotoxicity by increasing the release of ROS [171]; in this regard, brain infiltration of CD4+ and CD8+ cells was observed in both brain of post-mortem human PD specimens and in mouse PD model [172]. Furthermore, Th2 and Treg cells, which physiologically have a neuroprotective role, during aging tend to decrease, thus potentially contributing to induce microglial activation and risk of neurodegenerative diseases [173]. Likewise, circulating neutrophils undergo phenotypic alterations with aging which lead to increased chance of their hyperactivation [174]; hyperactivated neutrophils are able to disrupt the BBB integrity and are today considered a blood biomarker of AD severity and PD pathology [118, 175, 176].

Interestingly, increasing evidence is also focusing on the role of gut microbiota in priming neuroinflammation and oxidative stress responses [177]. Gut microbiota homeostasis strictly depends from peripheral immune system; indeed, microbiota-released chemokines and TLRs may act on peripheral immune cells and cause phenotypic changes of host mononucleate cells, thus contributing to inflammaging, neuroinflammaging and neurodegeneration [177]. Dysregulation of gut microbiota is considered necessary for the infiltration of peripheral immune cells in the brain, and is today recognized as a fundamental factor in the pathogenesis of neurodegenerative diseases [178].

## 4. Methodological advances in omics approaches: implication for neuroinflammation and neurodegenerative diseases

The recent methodological advances in omics approaches, including genomics, transcriptomics, metabolomics, lipidomics, and proteomics, have revolutionized the research field, allowing to generate large data sets, which represent a promising tool to greatly enhance the understanding of the molecular complexity of age-related neuroinflammatory and neurodegenerative diseases. Epigenomic studies employ single- or multi-omics approaches in order to further explore the broader mechanisms and interactions in biological systems in an unbiased way, instead of focusing on single molecules, in order to identify further risk factors linked to neurodegeneration [179].

### 4.1 Genome-wide studies (GWAS)

Genome-wide association studies (GWAS) represent a widely used research approach aiming at surveying the genomes of a a large group of individuals to identify genomic variants that are statistically associated with disease risk [180]. Despite a large portion of genetic variance remains still unexplained, GWAS and meta-analyses have recently showed a shared genetic architecture between AD and gastrointestinal tract disorders [181] again supporting the role of gut microbiota in Alzheimer’s disease pathogenesis, as well as the presence of genome-wide significant association signals in PD patients [182]. Furthermore, environmental and genetic factors were shown to interact to increase the risk for neuroinflammation, thus highlighting the importance of maintaining tight control over these molecular processes, to prevent disease progression [102]. Finally, it is noteworthy that many genes associated to increased AD risk encode for proteins implicated in glial activation and neuroinflammation, like for instance BIN1, CLU, TREM2, MSH6A or CD33 [183].

### 4.2 Epigenetic profile studies

More recently the emerging field of neuroepigenetics has expanded massively and it is now clear that neuroepigenetic mechanisms are key players in modulating the susceptibility to age-related diseases including AD and PD. In particular, it has been reported that several genes fundamental to the development of neurodegenerative disorders are deregulated by aberrant methylation patterns of their promoters, and even common epigenetic signatures for some dementia-associated pathologies have been identified. It has been hypostasized that most of these epigenetic changes occur over time in response to environmental effects and their accumulation can result in an increased risk of dementia, even if individually these changes might be essentially latent. This theory, known as epigenome-based latent early-life associated regulation (LEARn) has been proposed by Maloney et al [184], and it has recently gained considerable support with the identification of important neuroepigenetic mechanisms in several forms of dementia and other age-related diseases. This model explains the etiology of the age-related diseases within a framework in which epigenetic alterations during the lifespan would disturb gene expression over the long term, in an accumulative way, acting as a ‘seed’ for the development of the disease.

Technologies able to investigate the epigenetic profile have recently reached the stage at which large-scale studies are becoming feasible. The increasing interest in epigenome-wide association studies (EWASs) has supported the development of a growing number of analytical tools and packages for the analysis of array methylation data. Several publicly available datasets from different age-related phenotypes (including the different form of dementia) have been so far produced. Exploiting this precious source of information, two types of promising methylation-based biomarkers of biological age have been identified: the epigenetic clocks and the epigenetic mutation load (EML) models. Both these methylation-based biomarkers have been demonstrated to perform well as biological age predictors in multiple age-related diseases including several forms of dementia. While epigenetic clocks refer to a calibrated set of methylation markers (CpG sites), whose methylation levels are used to estimate chronologic or tissue age (programmed aging), in contrast epigenetic drift refers to a stochastic process that involves both gains and losses of the methylation state of CpG dinucleotides over time. Interestingly, since this process relies on the accumulation of these so-called stochastic epigenetic mutations (SEMs) occurring over the lifespan, it well fits with the previous mentioned LEARn theory. Understanding the epigenetic mechanisms that are altered in dementia, especially those associated with the initial phases, might allow not only to better clarify the etiopathology of dementia and its progression but also to design effective therapies to reduce this global public health problem.

### 4.3 Transcriptomic studies

Another promising omics approach to study age-related diseases is represented by transcriptomic methods, mainly consisting in sequencing and analysis of gene transcripts. In AD patients, the strongest transcriptomic changes have been already found at early phases of pathology, while upregulation of genes involved in the global stress response such as many TLRs, TNF and NFκB, was found at late stages [185, 186]. In a recent study, accelerated key aging-related transcriptome alterations have been detected in microglia and oligodendrocytes of AD patients [84].

However, transcriptomic analyses do not represent an accurate indicator of protein levels, which are always regulated by post-transcriptional events and are only partially correlated with RNA. Consequently, further studies are needed in order to integrate transcriptomics with proteomics.

### 4.4 Proteomic studies

Proteomics, represented by t*he analysis* of proteins and proteomes, mainly consisting in the separation, identification and quantification of the entire protein complement expressed by a genome, also allows to study the interactions among proteins involved in specific signaling pathways, metabolic pathways, and cellular processes [187, 188].

Neuroinflammatory proteins can be assessed in blood, cerebrospinal fluid, and post-mortem brain tissue [187, 188]. Proteomic analysis of single-cell immune profiling may help to better characterize the immune activity contributing to neuroinflammation and identify single-cell functional phenotyping that may reveal unique findings of immune dysfunction in neurodegenerative disorders. Until now, several protein biomarkers have been found altered at early stages of neurodegenerative diseases, mainly in AD and PD [189]. Four of them, namely cerebrospinal fluid amyloid-beta 42, amyloid-beta 42/40, total tau and hyperphosphorylated tau levels, have been incorporated in the recent diagnostic criteria for AD; other biomarkers such as the neurofilament light chain, which are associated with axonal damage, and the glia fibrillary acid protein, which is a biomarker of astrocytic activation, seem to be very promising but are not routinely used in clinical practice so far [190].

However, despite proteomics is an excellent technique for profiling inflammatory proteins in tissues and cells, to date, targeted approaches are still necessary for detecting peptides with very low concentrations. In addition, a deepest knowledge of the inflammatory profiles of peripheral and CNS resident immune cells is required to reveal novel mechanistic aspects of neurodegenerative diseases.

### 4.5 Metabolomic studies

The knowledge of interactions between individual lipid species or metabolites with other lipids, proteins, and metabolites, may add crucial information on disease regulation mechanisms. A recent study aimed to investigate short-term effects of systemic inflammation on the mouse plasma and brain cortical and hippocampal metabolome, lipidome, and proteome, revealed that neuroinflammation is associated with brain region-specific changes of metabolic pathways and lipids [191]. Furthermore, a metabolomic study based on cerebrospinal fluid analysis through nuclear magnetic resonance spectroscopy found a typical metabolomic fingerprinting able to differentiate with good accuracy patients with AD and controls. This investigation also reported reduced cerebrospinal fluid levels of acetate, a marker of glial metabolism, in AD patients compared to controls [192]. However, since many metabolites are below the limit of detection/quantification of current analytical protocols and/or are not present in spectrum libraries, their analyses still represent a challenge.

In conclusion, current multi-omics integration methods can be classified into two general categories: statistical integration and network-based integration. Statistical integration relies on drawing inferences from the data themselves without using prior knowledge. Network-based integration, on the other hand, views biological systems as interconnected entities, with each omics contributing to revealing the true connections of the networks. Altogether, multi-omics integration for age-related neuroinflammatory diseases is still developing and requires further studies and applications.

## 5. Discussion

Ageing is typically associated with neuroinflammation, now termed “neuroinflammaging”, a process recognized as the main risk factor and/or a direct contributor of the occurrence of neurodegenerative disorders [5].

Neuroinflammation is hypothesized to develop from chronic immune system activation in the brain in response to a plethora of stressors/insults fueling the activation and invasion of the brain by circulating immune cells and/or their secreted molecules. The production of toxins that result in the death or malfunctioning of neurons in neurodegenerative illnesses is significantly modulated by age-dependent heightened neuroinflammatory processes. In other words, inflammatory levels may represent a sort of tine borderline between physiological brain aging and pathological brain aging.

### 5.1. Clinical and therapeutic implications

A deeper understanding of mechanisms and biomarkers of neuroinflammaging holds many potential clinical implications. Indeed, global population aging is currently leading to a striking increase in the prevalence of dementia and age-related cognitive disorders [193, 194], with increased social and economic burden on healthcare systems. This is mainly due to the absence of therapeutic approaches capable of curing neurodegenerative diseases, so that, after symptom onset, it is often too late to reverse the underlying mechanism of the pathology. In this regard, addressing neuroinflammaging, by counteracting detrimental proinflammatory processes and increasing anti-inflammatory responses on a global scale, may be the key to preventing neurodegenerative disorders and decreasing the social and economic burden on individuals and societies [195]. In recent years, healthy lifestyle is emerging as a promising non-pharmacological approach to delaying human and brain aging [195, 196]. Several epidemiological studies have shown that meta-inflammatory responses due to excessive consumption of refined sugars, red meat, as well as saturated fats can stimulate neuroinflammation and neurodegeneration [197]; similarly, obesity and metabolic disorders related to insulin resistance like diabetes mellitus increase the risk of neurodegenerative disorders [198, 199]. To counteract these unhealthy habits and metabolic derangements, several nutritional strategies, including caloric restriction, intermittent fasting and specific nutrition patterns (e.g. vegetarian and Mediterranean diet) have shown benefits and protective effects on the brain, also when started in mid- or late-life [196, 200]; in particular, specific dietary patterns including vegetarian diet and Mediterranean diets, based on consumption of vegetables, fish, fruit, and low-fat dietary, were reported to slow cognitive decline in humans, also acting by epigenetic modifiers [201-204]; similarly, intermittent fasting seems to be capable to affect circadian clock gene expression in human blood cells with subsequent increase in production of SIRT-1 and BDNF and enhanced neuroprotection[205]. However, it is still not clear that these reported effects are related by intrinsic properties of intermittent fasting or are mediated simply by reduction of food intake regardless of the diet scheme.

The neuroprotective effects related with physical and cognitive exercise are also emerging as potential approaches to decrease neuroinflammaging. In animal studies, anaerobic exercise was reported to reverse BDNF mRNA induction and to increase microglial expression of IGF-1, which is known to be neuroprotective but generally decreases with age [206]; moreover, aerobic exercise was shown to improve learning memory and neuroplasticity in old individuals [207], with higher effects when combined with cognitive stimulation [208].

Balancing nutrition and exercise may thus represent a useful approach to try to reduce the risk of age-related cognitive decline and neurodegenerative disorders by modulating neuroinflammaging pathways and neuroepigenetic mechanisms.

### 5.2. Limitations and controversies of targeting neuroinflammaging

One of the main challenges in appropriately targeting molecular pathways of neuroinflammaging is represented by differentiation of beneficial from detrimental neuroinflammaging processes. Despite the growing amount of research in this field, to date a fundamental question remains unsolved: why, if in almost all elderly people the signs of neuroinflammaging, can be found, only some will be affected by neurodegenerative diseases? The discrimination of the genetic and environmental factors determining the allocation of aged people in one of the two categories (healthy old subjects vs aged patients affected by neurodegenerative diseases) is the crucial challenge that researchers must face at this moment. Indeed, if one hand we already know many mechanisms underlying inflammaging and neuroinflammaging, on the other hand we are not still able to recognize which aged individuals will be destinated to develop age-related neurodegenerative diseases and which not. The first hypothesis to explain this dilemma is that people destinated to reach extreme age in relatively good health have got protective anti-inflammatory epigenetic and genetic factors that act against the deleterious effects of neuroinflammaging [209, 210]; at a brain level, recent studies have shown that centenarian populations tend to be more resilient and less prone to cognitive impairment, even when exposed to cognitive risk factors[211]. Those individuals, despite having high levels of peripheral pro-inflammatory cytokines, possess also strong anti-inflammatory phenotypes, probably involved in delay of disease onset, preservation of cognitive reserve, and prolongation of life span [212]. Neuroprotection in particular seems to be associated to activation of the repressor element-1-silencing transcription (REST) which is able to down-regulate genes involved in neuronal excitation and synaptic functioning [213]. The second hypothesis is based on the existence of individual-specific critical thresholds beyond which neuroinflammaging may induce neurodegeneration, but until now we do not make an exact *in vivo* quantitative assessment of the multiple and often interconnected neuroinflammatory processes typically associated with age.

Another fundamental point is to understand why some people develop sporadic (i.e., not hereditary) forms of neurodegenerative disorders under 65 years of age. A hypothesis is that in these people neuroinflammatory processes associated with aging are accelerated because of genetic and epigenetic factors; another one is that unknown mechanisms other than neuroinflammaging are the main determinants of neurodegenerative processes, at least in these people.

**Table 1:** Epigenetic changes associated to increased protein marker detection.

Epigenetic changes	Cytokine increased levels	Scores*
**DNA transmethylation** **Global hypomethylation**	IL-1β, TNFα, IL-6	------
**DNA methylation combined with acetylation**	TNFα	------
**H3 acetylation** **Monoubiquityl at Lys 10 of H2b** **Phosphorilation H3 Serine 10**	NF-κβ	------
**miRNA2,** **miRNA146a** **miRNA195** **miRNA221**	TNFα, IL-1β, TNFα, IL-6, NF-κβ TNFα, IL-1β, TNFα, IL-6, NF-κβ, TRAF6 TNFα, IL-1β, iNOS (indirect effect)	------

Blood proinflammatory and epigenetic markers are useful to regularly monitor aged people.

* Each score should be assigned by setting up ranges, according to association studies, and the value can be determined according to the increased levels. For example, if NF-κβ and H3 acetylation show increased value of 50% respect the normal levels, in a range score from 1 at 10, the value is 5.

In our opinion, both epigenetic and multiomic research may be efficacious ways to answer these crucial questions and to personalize treatments targeting neuroinflammaging in high-risk patients according to principles of precision medicine. Indeed, a one-size-fits-all approach, based on decreasing neuroinflammaging cascades without quantifying their magnitude and without considering the specific underlying conditions of each patient, may harmfully contrast physiological pathways which allow individuals to respond to internal and external stressors, thus increasing the risk of adverse events and death. This was partly highlighted in the CANTOS Trial in patients with atherosclerotic disease: targeting IL-1β-mediated pathway through administration of canakinumab had the beneficial effect of significantly decrease major adverse cardiovascular events, but with increased risk of fatal infections compared with placebo [214]. In the case of neuroinflammaging, the clinical and biological complexity is even higher, given that patients with cognitive impairment are often frail, and more vulnerable to adverse events and to developing dementia [215, 216].

The solution of these complex problems may be strategic both in the view of early diagnosis and preventive therapeutic interventions. To achieve this, the adoption of “red flags checklists”, containing specific clinical indicators of possible serious underlying neuroinflammatory conditions could be helpful. More in detail, regular screening of aged people could be performed by detecting and quantifying the presence of specific epigenetic and inflammatory markers in peripheral blood. In order to capture the interindividual variability in neuroinflammaging pathways, a personalized approach based on omics should be applied to each subject, but the cost of such approach is still very high [217]. Alternatively, we suggest the screening of some so-called sentinel markers, such as specific pro-inflammatory cytokines, DNA hypomethylation and histone modifications [218-220]. Each assessed biomarker should be assigned a score, thus setting up ranges identifying distinct risk classes with distinct monitoring frequencies (Table 1).

For the exogenous factors, such as those listed in Table 2, the score should be calibrated according to the age, the exposure rate, the documented association with epigenetic mutations and inflammatory indices.

**Table 2:** Exogenous risk factors associated with time of exposure.

Risk factors	Time of exposure	Scores*
**Particulate matter** **Black carbon** **O3** **Organic pollutants**	---------	---------
**Ionizing radiation exposure**	----------	----------
**Microbial exposure**	----------	----------
**Chronic infections**	----------	----------
**Nutritional deficiences**	-----------	-----------
**Zn deficiency**	-----------	-----------

* Each score should be assigned by setting up ranges, according to association studies and the time of exposure, and the value can be determined according to the increased levels. For example, if the time of exposure is for the 50% of the average life, the selected value is 5 in a range score from 1 at 10- This checklist is incomplete, since it does not consider all exogenous risk factors and the rate exposure.

### Conclusions

In conclusion, there is the urgent need to further improve the current knowledge concerning the link between neuroinflammaging and neurodegenerative disorders, to address chronic inflammation without compromising acute defenses against pathogens and stressors.

Epigenetic modifications have proven to be extra ordinary sources of robust and reproducible biomarkers of aging and age-related phenotypic traits, more stable than RNA or protein-based biomarkers. Lastly, the current thrive of epigenetic drugs and interventions will soon provide new strategy to combat neuroinflammaging, but new pharmacological treatments need to balance the relationship between risks and benefits. For this aim, identification of signature biomarkers to distinguish detrimental neuroinflammaging from acute stress responses will be necessary to optimize treatment plans in high-risk individuals.
